# Evaluating the spatiotemporal patterns of drought characteristics in a semi‐arid region of Limpopo Province, South Africa

**DOI:** 10.1007/s10661-024-13217-6

**Published:** 2024-10-17

**Authors:** Selelo Matimolane, Sheldon Strydom, Fhumulani Innocentia Mathivha, Hector Chikoore

**Affiliations:** 1 Unit for Environmental Sciences and Management, North-West University, Potchefstroom, South Africa; 2 Equitable Education and Economies, Human Sciences Research Council, Pretoria, South Africa; 3 Department of Geography, Rhodes University, Grahamstown, South Africa; 4 Department of Water and Sanitation, University of Limpopo, Sovenga, South Africa; 5 Department of Geography and Environmental Studies, University of Limpopo, Sovenga, South Africa

**Keywords:** Climate change, Drought indices, Reconnaissance Drought Index, Potential evaporation, Semiarid environments

## Abstract

Drought is a complex phenomenon resulting from below-average rainfall and is characterized by frequency, duration, and severity, occurring at a regional scale with dire consequences, especially in semiarid environments. This study used the Reconnaissance Drought Index (RDI) to assess drought severity in two district municipalities in Limpopo Province. Rainfall and air temperature data from 12 stations covering 1970–2020 were obtained from the Agricultural Research Council. The calculation of RDI relies on the monthly accumulation ratio of total rainfall to potential evapotranspiration (PET). For this study, PET was estimated using the Hargreaves and Samani temperature-based approach. The RDI results showed a high spatial–temporal variation in drought characteristics over the study area. All stations experienced extreme drought conditions in different years, with the maximum drought severity (-3.40) occurring from 2002–2003 in the western parts of the study area, indicating extreme drought. Furthermore, the results revealed continuous drought conditions over various periods, including severe droughts between 1995 and 1998 and between 2014 and 2016, with the severity varying between mild and moderate drought conditions. The results reveal notable but nonuniform drought patterns as the climate evolves, with potential implications for water availability and livelihoods. The study's findings underscore the significance of adopting multidimensional approaches to drought assessment that encompass meteorological and hydrological factors to inform strategies for adaptive water management and policy formulation in the face of a changing climate.

## Introduction

Drought is recognized as a widespread natural occurrence characterized by a deficiency of water resources across extensive geographical regions and presents a complex challenge across the world (Orimoloye et al., 2022; Vangelis et al., 2013). Arguably, drought is a concept that relies on a relative perspective, where the shortfall in rainfall needs to be assessed considering the usual climatic patterns of precipitation and all activities related to water resources. Depending on the geographic location, its duration may span weeks, months, or even years (Chivangulula et al., 2023; Orimoloye et al., 2022). According to Wilhite and Glantz (1985), drought is an evolving phenomenon initiated by insufficient rainfall, and it can be categorized as meteorological, hydrological, agricultural, or socioeconomic. The initial three categories relate to the physical hydrological cycle, in contrast with socioeconomic drought, which considers drought in relation to supply and demand, monitoring the repercussions of water scarcity as they propagate through socioeconomic frameworks. The onset of drought is characterized by a prolonged and significant shortage of water, both in terms of precipitation and available water resources (Chikoore, 2016). This stage of a drought event can be identified as a meteorological drought, where there is a discernible departure from the normal or expected climatic conditions related to precipitation levels, a decrease in rainfall, and a gradual decline in water levels in rivers and reservoirs. Higher global warming is projected to increase the probability of compound extremes such as concurrent heatwaves and droughts, compound flooding, and fire weather (IPCC, 2023) leading to severe impacts in developing regions with low adaptive capacity such as southern Africa.

Drought events have devastating impacts beyond meteorological impacts, influencing hydrological, agricultural, ecological, and socioeconomic systems. The cascading effects of meteorological drought can lead to agricultural drought (Chivangulula et al., 2023), characterized by insufficient soil moisture leading to crop loss. In severe cases, droughts tend to have far-reaching consequences which negatively affect socioeconomic activities. In the worst scenarios of drought impacts, various sectors and aspects of society are significantly affected, leading to a complex web of challenges. Due to its nature, the failure to detect drought events and implement effective mitigation measures will inflict considerable harm on the community's livelihoods and cause negative impacts on the economies of the impacted regions (Naumann et al., 2014). Drought also impacts the economy, crop yields, number of herds, increased fire risk, and rural livelihoods (Chikoore, 2016; Nembilwi et al., 2021; Singo et al., 2023; Vicente-Serrano et al., 2012).

Drought indices offer a systematic and standardized approach to summarizing complex climate data, facilitating the identification of long-term climate trends, anomalies, and extremes (Gallant & Karoly, 2010). Notably, various indices have been developed (Abubakar et al., 2020; Botai et al., 2016; Hasan et al., 2023; Hoffmann et al., 2020), and they play a crucial role in understanding the severity, duration, and spatial extent of drought events. Among the commonly employed meteorological drought indices are the Palmer Drought Severity Index (PDSI) (Palmer, 1965), Deciles Index (Gibbs & Maher, 1967), Rainfall Anomaly Index (Van Rooy, 1965), Standardized Precipitation Evapotranspiration Index (SPEI) (Vicente-Serrano et al., 2012), Standardized Precipitation Index (SPI) (McKee et al., 1993), Reconnaissance Drought Index (RDI) (Tsakiris & Vangelis, 2005), and Drought Area Index (Bhalme & Mooley, 1980). Although not as sophisticated as the PDSI, RDI estimates water deficit based on precipitation as well as evaporation (Tsakiris & Vangelis, 2005), making its formulation better than the widely applied SPI which relies on precipitation to estimate water deficit.

Drought indices vary in complexity and application, allowing for adaptability to different climate conditions and regions. To address the drawbacks of the SPI, studies have reported that it is crucial to evaluate meteorological drought using the RDI under both high- and medium-emission scenarios and conditions (Sayari et al., 2013; Yisehak et al., 2021). Chikoore (2016) noted that the SPI does not account for the impacts of air temperature, soil moisture, or evapotranspiration, which are essential components of the surface water balance. Due to the uncertainty associated with drought severity assessment, Jain et al. (2015) recommended the integration of drought indices, encompassing the entire hydrological cycle, and other factors to improve their reliability. Abubakar et al. (2020) used the RDI to assess drought characteristics in Free State Province, which further supports that the RDI could serve as a valuable index for appraising the impact of drought on agricultural production. Similarly, Mohamed et al. (2022) used RDI to measure drought severity and analyze the variability of drought events in Somalia during the last decade.

Limpopo Province is prone to severe climate events and is characterized by elevated evaporation rates and exceptionally arid conditions, frequently resulting in meteorological droughts (Botai et al., 2016). According to Du Plessis and Schloms (2017), this increase is further exacerbated by unpredictable rainfall patterns, averaging approximately 465 mm (Du Plessis & Schloms, 2017), and a high frequency of dry spells (Usman & Reason, 2004). The remote El Nino phenomenon is often associated with drought events (Chikoore & Jury, 2021; Mathivha et al., 2024) and heat waves in this region (Mbokodo et al., 2023).

Most drought studies are focused on regional and national scales, with fewer conducted at the local level, even though it is at the local scale where predictions are most crucial. Given the increasing concern about the influence of climate change on the frequency and severity of drought events in semiarid regions, drought assessment studies conducted using a range of different indices are important. Although similar studies have been carried out in the region (Chikoore & Jury, 2021; Mathivha et al., 2024; Nembilwi et al., 2021), these studies made use of standardized indices as well as PDSI. Therefore, along with updating the existing drought analysis literature, this study will address the gap in research within the region by applying the RDI to assess meteorological drought characteristics and to identify significant trends in RDI. over a long-term period. This is significant because, as previously revealed, this region is prone to drought events that negatively impact water availability and disrupt the livelihoods of local communities. Thus, this study aimed to characterize the spatiotemporal patterns of drought occurrence and severity.

## Materials and methods

### Description of the study area

**Fig. 1:**
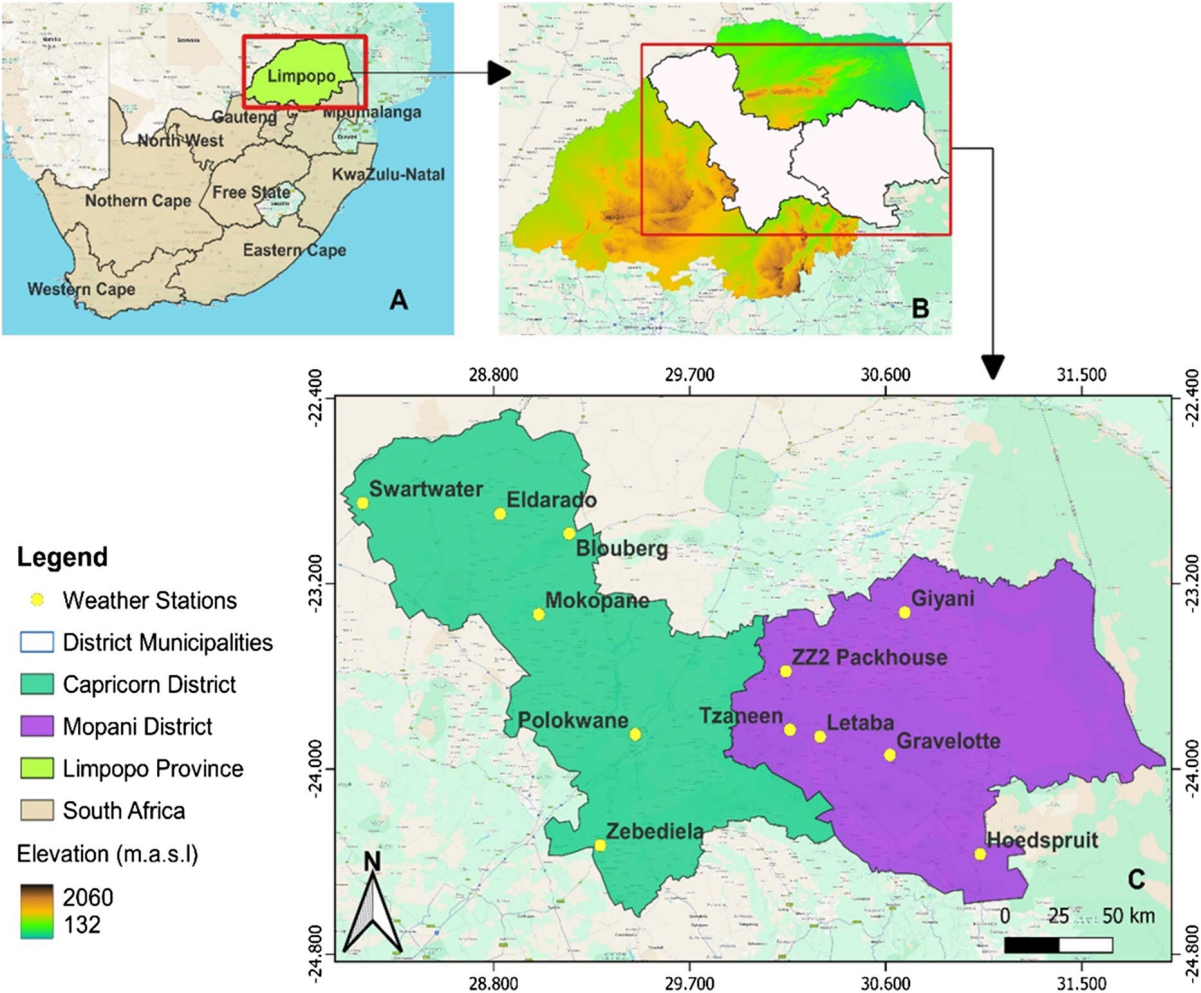
Study area map showing (**A**) The Limpopo Province in relation to South Africa, (**B**) The Capricorn and Mopani district municipalities within the Limpopo Province, and (**C**) The locations of the selected weather stations in the two district municipalities which are shown by the yellow dots

The study was conducted in the Capricorn District Municipality (CDM) and Mopani District Municipality (MDM) in Limpopo Province, which is located in the northernmost part of South Africa (Fig. 1). The province occupies an area of approximately 125,754 km^2^ with the highest elevation of 2126 m, topographically dominated by isolated mountains (Matimolane et al., 2023). The CDM consists of four (4) local municipalities, namely, Blouberg, Molemole, Polokwane, and Lepelle-Nkumpi. The district has a total area of 21 705 km^2^, which constitutes 12% of the total area of the province (CDM IDP, 2022). The MDM is situated in the northeastern part of the province and covers a total area of 20 011,0 km^2^ (IPD 2022/23) with five local municipalities; Maruleng, Greater Tzaneen, Greater Letaba, Greater Giyani, and Ba-Phalaborwa are bordered in the north by Vhembe District Municipality (IPD 2022/23).

The study area has a climate that spans from subtropical to semiarid (Fig. 1C). In the western regions, there is a dry, hot semiarid climate, transitioning to cooler conditions during the escarpment. The summer precipitation over southern Africa is of convective origin forced by large-scale dynamics. ENSO influences interannual rainfall variability, of which drought is dominant during the El Niño phase in austral summer, while above-normal rainfall conditions are associated with La Niña (Rouault et al., 2024). The Luvuvhu/Letaba area is marked by subtropical temperatures and elevated humidity (Fitchett et al., 2016). Significant seasonal temperature fluctuations are evident, with the eastern sections of the study area, such as Polokwane, generally experiencing cooler temperatures than the northeastern parts, as exemplified by Giyani. January typically has peak maximum temperatures, while minimum temperatures are, on average, recorded in July (Holland, 2011). Certain parts of the province are deemed highly suitable for agriculture, benefiting from a sufficient water supply for irrigation. Much of the rainfall, more than 80%, occurs from late September to March, occasionally continuing into early April. The average annual rainfall varies from 500 to 800 mm, with a mean average annual temperature between 20 °C and 22 °C (Petja et al., 2014).

Economically, the key sectors in the study area include agriculture, manufacturing, mining, and community services. The agricultural sector, especially the agro-processing of tomatoes, citrus fruit, and maize production, holds significant potential and plays an important role in the district’s economy. The tourism sector has also been identified as an economic driver (Capricorn District Municipality Integrated Development Plan, 2022). The district comprises diverse landscapes with forests, cultural villages, dams, art, and monuments. Over 85% of the domestic tourists visiting the districts are for leisure and adventure (Myer & De Crom, 2013).

### Dataset collection

Meteorological data, including rainfall, maximum air temperature, and minimum air temperature, from 1970 to 2020 were freely sourced from the agrometeorology database of the Agricultural Research Council (ARC) at 12 weather stations (Table 1). The ARC Agrometeorology Programme maintains an operational national agro-climate network of weather stations (approximately 500) covering all distinct geographical regions (https://www.arc.agric.za/arc-iscw/Pages/Climate-Monitoring-Services.aspx). The quality and availability of meteorological data are critical for various applications, including weather forecasting, climate research, and disaster management. The selected stations are distributed and cover the different climatic zones of the study area (Fig. 1). Weather station networks may have gaps in coverage, particularly in sparsely populated or remote regions. These gaps can limit the accuracy of regional weather forecasts and climate monitoring. Thus, only stations with continuous data exceeding 90% were chosen; however, certain stations had missing data and inconsistencies. To address the gaps in the data, we utilized the ARC standalone patching tool in this research, applying the multiple linear regression technique detailed by Shabalala et al. (2019).

**Table 1:** Selected weather station characteristics

Station Name	Latitude (° S)	Longitude (° E)	Period	Altitude (m)	Mean Minimum Temperature (°C)	Mean Minimum Temperature (°C)	Annual rainfall (mm)
Polokwane	-23.850	29.450	1970 – 2020	1242	25,20	11,82	808,97
Zebediela	-24.329	29.289	1975 – 2020	1053	27,78	12,11	782,97
Blouberg	-22.984	29.147	1980 – 2020	852	28,72	13,74	522,37
Mokopane	-23.332	29.007	1985 – 2020	827	27,42	13,69	665,22
ZZ2-Packhouse	-23.578	30.141	1986 – 2020	671	26,58	11,93	466,21
Swartwater	-22.852	28.199	1989 – 2020	830	31,26	14,85	483,69
Eldorado	-22.898	28.830	1990 – 2020	814	30,23	14,08	648,38
Tzaneen	-23.830	30.160	1980 – 2020	726	27,74	15,35	986,38
Letaba	-23.860	30.298	1985 – 2020	865	23,62	8,73	576,46
Gravelotte	-23.939	30.619	1989 – 2020	590	29,78	15,32	478,38
Giyani	-23.324	30.687	1990 – 2020	460	30,45	15,29	484,34
Hoedspruit	-24.367	31.033	1990 – 2020	500	28,83	15,99	455,49

## Methods of analysis

### Estimation of evapotranspiration

The DrinC software for drought analysis includes an incorporated unit for computing PET using temperature-based techniques, such as those proposed by Hargreaves and Samani (1982, 1985). For this study, the Hargreaves and Samani equation, which is renowned for its simplicity, has been extensively applied in semiarid and arid regions (Lang et al., 2017) to estimate reference evapotranspiration, as expressed below:

1 $$PET=0.0023{R}_{\alpha } \left(T+17.8\right) {({\text{T}}_{\text{max}}-{\text{T}}_{\text{min}})}^{0.5}$$

In this context, PET represents the calculated reference evapotranspiration; ${R}_{a}$ corresponds to the water equivalent of extraterrestrial radiation, calculated following the procedures described by Allen et al. (1998). A proposed alternative is used in this study, i.e., the Hargreaves equation, which is based on values of ${\text{T}}_{\text{max}}$ and ${\text{T}}_{\text{min}}$ only (Raziei & Pereira, 2013). This method has been shown to provide better estimates of Eto compared to Thornthwaite and Penman–Monteith (Moeletsi et al., 2013), particularly under hyperarid and arid conditions. Thus, ${\text{T}}_{\text{max}}$ (°C), ${\text{T}}_{\text{min}}$ (°C) air temperature and T denote the daily maximum, minimum, and mean air temperature (°C), respectively, where T is calculated as the average of ${\text{T}}_{\text{max}}$ and ${\text{T}}_{\text{min}}$. The initial empirical coefficient, established by Hargreaves and Samani (1985), is 0.0023. It is worth noting that the Hargreaves and Samani equation provides an estimate of PET using minimal meteorological data, primarily temperature (Gavilán et al., 2006). However, it has proven especially useful in situations where more comprehensive meteorological datasets are not readily available. The problems associated with selecting an appropriate time scale for analyzing data to understand drought severity highlight the importance of investigating the impact of PET trends and patterns on incidents and the severity of drought (Gidey et al., 2018). To this point, other studies have demonstrated that indices that use monthly precipitation are straightforward to employ since they use the total values of meteorological variables over a month (Abubakar et al., 2020; du Plessis & Schloms, 2017; Dunkerley, 2019).

### Reconnaissance Drought Index

The severity of the drought was evaluated by RDI (Tsakiris & Vangelis, 2005; Tsakiris et al., 2007a) using Drought Indices Calculator (DrinC software) software (available here: https://drought-software.com/) (Tigkas et al., 2015). The software package was developed by Tigkas et al. (2015) at the University of Athens to provide a comprehensive, simple, and adaptable interface for the calculation of drought indices. RDI has recently garnered significant recognition, particularly in arid and semiarid climatic regions, as a robust drought index due to its reliance on both rainfall and PET (Abubakar et al., 2020; Imzahim et al., 2019; Thomas et al., 2016). Singer et al. (2021) considered PET as a theoretical evaporative demand, which is required to calculate actual evapotranspiration. By considering PET, RDI accounts for the demand for water by plants under prevailing meteorological conditions, offering a more realistic depiction of drought stress. Additionally, RDI has low data requirements and high sensitivity, making it particularly suitable for arid and semiarid climates where water scarcity is a critical concern (Asadi-Zarch et al., 2011; Halwatura et al., 2017; Mohammed & Scholz, 2017; Wang et al., 2015).

In this study, Monthly RDI was calculated using the monthly precipitation and evapotranspiration data of 12 stations in Limpopo Province. SPI and RDI values were calculated in the period of hydrological years from 1970–71 to 2019–2020 for the time scale of 12 months. In arid and semi-arid regions, drought events assessed over extended periods may appear to occur with the same frequency across all locations due to index standardization ((Shamsnia, 2014). Additionally, in regions with high precipitation, SPI values calculated at short time intervals might produce deceptively large negative or positive results (Shamsnia, 2014). In contrast, in arid and semi-arid regions, high temperatures combined with low precipitation are key factors in the development and intensification of droughts (Thomas et al., 2016). RDI is particularly suited for identifying meteorological drought characteristics in these regions and has been widely used by researchers (Thomas et al., 2016).

In its standardized form, the index can favorably be compared with other indices, providing a basis for cross-validation and a robust evaluation of drought severity (Wang et al., 2015). RDI can be applied at various spatial scales, from local to regional, and at diverse temporal scales (such as monthly, seasonal, or annual). This flexibility and use of consistent methodologies for calculating RDI allows for in-depth spatial and temporal analyses of drought, providing room for meaningful comparisons and trend analyses over time. It is important to note that the calculation of RDI may be adapted based on regional characteristics, available data, and specific research requirements. Additionally, some studies (Katipoğlu et al., 2020; Mohamed et al., 2022) have used variations in the RDI formula to enhance its applicability to different climates and conditions for calculating the RDI based on established methodologies. Tsakiris and Vangelis (2005) and Tsakiris et al. (2007b) suggested that the RDI formula can be expressed in three ways, namely, alpha (${\text{RDI}}_{\alpha k}$), normalized (${\text{RDI}}_{\text{n}}$) and standardized (${\text{RDI}}_{\text{st}}$) forms. The initial value ${\alpha }_{k}$, displayed in aggregated form with a monthly time step can be computed for the entire hydrological year or each month alone, and the cumulative PET time scale αo is typically computed annually for the *i-th* year using Eq. (2).

2 $${\alpha }_{k}=\frac{\sum_{\text{ J}=1}^{12}{\text{R}}_{\text{ij}}}{\sum_{\text{ j}=1}^{12}{\text{PET}}_{\text{ij}}}, i=1\left(1\right)N and j=1\left(1\right)k$$

where *N* is the total number of years in hydrological year *i* and ${R}_{ij}$ and ${PET}_{ij}$ are the rainfall and the potential evapotranspiration of the *j-th* month of the *i-th* year, respectively. The values of ${\alpha }_{k}$ were evaluated across a wide range of places and periods, and they properly followed both the gamma and lognormal distributions. *N* is the total number of years in hydrological year *i* and in the *-th* year. For this paper, ${\text{RDI}}_{\text{st}}$ was adopted, as documented in Vangelis et al. (2013) and Khajeh et al. (2019), and its strength lies in drought severity assessment and is considered reliable. The ${\text{RDI}}_{\text{n}}$ was applied in this study for comparative purposes using Eq. (3). Mohammed (2021) reported that by using the ${\text{RDI}}_{\text{n}}$ form, more consistent climate variable trends can be identified than by applying PET and rainfall time series separately.

3 $${\text{RDI}}_{\text{n}}\left(\text{k}\right)=\frac{{a}_{\text{k}}}{{\overline{a} }_{k}}-1$$

where ${\overline{a} }_{k}$ is the long-term average of ${a}_{\text{k}}$. Assuming that the values of ${a}_{\text{k}}$ follow a log-normal distribution, the standardized form of the index is computed using Eq. (4).

4 $${\text{RDI}}_{\text{st}}(\text{k})=\frac{{\text{y}}_{k}-{\text{y}}_{\text{k}}^{-}}{{\widehat{\upsigma }}_{k}}$$

where ${\text{y}}_{\text{k}}$ is the natural logarithm of $\text{ln}{\alpha }_{\text{k}}$, ${\overline{\text{y}} }_{\text{k}}$ is its arithmetic mean, and ${\widehat{\upsigma }}_{\text{k}}$ is the standard deviation. Relative to the research area's normal conditions for the time under consideration, positive values for α indicate uncharacteristic wet periods and negative values indicate dry periods. Accordingly, as shown in Table 2, we can categorize drought severity into four groups: mild, moderate, severe, and extreme (Wang et al., 2019). The corresponding drought levels are denoted by α. This is significant because categorizing drought contributes to understanding its characteristics, impacts, and appropriate mitigation strategies. Different types of droughts may coexist or transition into one another depending on various factors, including climate, geography, and human activities.

**Table 2:** Establishment of RDI drought levels (Tigkas et al. 2013)

Drought level	Drought class	Range of RDI occurrence
1	Extreme Drought	≥ -2.00
2	Severe Drought	-1.50 to -2.00
3	Moderate Drought	-1.00 to -1.50
4	Mild Drought	-0.5 to -1.00
5	No Drought	- ≤ 0.5

### Principal Component and Trend Analysis

Exploratory principal component analysis (PCA) was performed using the FactoMineR package for computation and multivariate exploratory data analysis (Lê et al., 2008). The factoextra Version 1.0.7 package for the PCA results in RStudio was used to visualize the variables (Kassambara & Mundt, 2020). PCA is a widely used statistical procedure for summarizing information in large datasets (Camargo, 2022). The PCA was computed based on the transformed and standardized means, standard deviations, coefficients of variation, aridity indices, and climatic conditions to explore patterns in the correlation matrix. The purpose of using PCA is primarily to reduce the dimensionality of large datasets and to identify patterns, trends, and dominant modes of variability within the climate data, such as changes in precipitation and temperature.

The Mann–Kendall (MK) and Sen’s slope estimator trend tests were used to assess the statistical significance of trends in annual climate extreme indices. Both the MK test and Sen’s slope estimator are nonparametric methods commonly employed by researchers studying hydrological and climate time series due to their robustness in handling missing data and nonnormally distributed datasets, which are common in hydrometeorological data analysis (Asare-Nuamah & Botchway, 2019; Rahman et al., 2022). Nonparametric tests are preferred in various studies because of their ability to manage such data characteristics. The MK test determines whether statistical trends are random without requiring prior knowledge of the data distribution (Yu et al., 2002). On the other hand, Sen’s slope estimator is considered more effective than traditional regression equations. Hence, this approach is deemed the most suitable method for analyzing trends in climatological time series across the eastern and western Limpopo Province. The MK trend test was conducted at a significance level of 0.05 for the RDI series to identify the presence of trends in the time series. The test statistic S is calculated using Eq. (5), while Sen’s slope estimator was employed to assess the magnitude of the trends (Yacoub & Tayfur, 2020).

where ${\text{X}}_{\text{j}}$ and ${\text{X}}_{\text{k}}$ are sequential data values for the time series data of length n and Sgn is obtained via Eq. (5).

5 $$\text{Sng }\left({X}_{j}-{X}_{k}\right)=\left\{\begin{array}{c}+1 if \left({X}_{j}-{X}_{k}\right)>0\\ 0 if \left({X}_{j}-{X}_{k}\right)=0\\ -1 if \left({X}_{j}-{X}_{k}\right)<0\end{array}\right\}$$

The average value of S is E[S] = 0. The value of the S statistic is associated with the test statistics $\uptau$, and this is estimated using Eq. (6).

6 $$\tau =\frac{\text{S}}{\text{n}(\text{n}-1)/2}$$

The test for the Sen slope estimator considers measurements *Y*_*1*_*, Y*_*2*_*, and Y*_*3*_*..., Yn* of time series taken at times *t*_*1*_*, t*_*2*_*, t*_*3*_*,..., tn*, where *t*_*1*_ ≤ *t*_*2*_ ≤ *t*_*3*_ ≤ *...* ≤ *t*_*n*_, as independent observations. The gradient *D*_*k*_, (*k* = *1,2,3,... N*), for each *N* pairs of observations taken at times *t*_*j*_ and *t*_*i,*_ such that *1* ≤ *i* ≤ *j* ≤ *n* and (* t*_*j*_—*t*_*i*_) > 0, can be calculated as:

7 $${D}_{k}=\frac{{Y}_{j}-{Y}_{i}}{{t}_{j}-{t}_{i}}$$

The estimate of trend ($\widehat{\beta }$) in the data series *Y*_*1*_*, Y*_*2*_*, and Y*_*3*_*..., Y*_*n*_ can then be calculated as:

8 $$\widehat{\beta }=\left\{\begin{array}{c}{D}_{(\frac{N-1}{2})+1}\;if\;N\;is\;odd,\\ \frac{{D}_\frac{N}{2}+{D}_{\left(\frac{N}{2}\right)+1}}{2}\;if\;N\;is\;even.\end{array}\right.$$

The above represents an empirical nonparametric calculation of the median of *D*_*k*_. The (1 − α) confidence interval for $\widehat{\beta }$ may be calculated as follows (Wang & Swail, 2001):

1) Compute M_1_ and M_2_ using the estimate (Vs) from the Mann‒Kendall test described above and the (1 − α⁄2) quantile of the standard normal distribution (*Z*_(1−α⁄2)_) as:

9 $${\text{M}}_1=\frac{(\text{N}-{\text{Z}}_{(1-\alpha/2)}{\text{V}}_\text{s}}2and\;{\text{M}}_2=\frac{(\text{N}+{\text{Z}}_{(1-\alpha/2)}{\text{V}}_\text{s}}2$$

2) Determine the order statistics *D*_*M*1_ and *D*_*M*2+1_ as the lower and upper (1 − α) confidence limits, respectively, from the collection of the N gradients (*D*_*k*_).

## Results and Discussion

### Calculated potential evapotranspiration

The results of the calculated PET (Table 3) show that the Swartwater, Giyani, Gravelotte, and Eldarado stations were under hyperarid climatic conditions with the highest monthly average PET (154.93, 151.18, 151.97, and 147.87 mm per month, respectively). Moreover, the findings of this study revealed elevated PET values across all stations. Consequently, in an optimal environment with all plants covering the ground, consistent plant height, leaf cover, and an abundant water supply, the water vapor flow would be greater than the precipitation that falls in the study region (Allen et al., 2020). This is substantiated by a noticeable trend of rising mean air temperatures over the past three decades, resulting in a significant (p < 0.05) increase in potential evapotranspiration across the stations (Table 4b).

**Table 3:** Monthly PET and climatic conditions of selected stations for 1970–2020

Station name	Mean PET (mm)		Standard deviation (mm)	Coefficient variation (%)	Aridity index	Climate condition
Giyani		151.18	10.63	7.03	0.01	Hyperarid
Gravelotte		151.97	10.45	6.88	0.01	Hyperarid
Hoedspruit		137.73	9.93	7.21	0.28	Semi-Arid
Letaba		125.50	10.53	8.39	0.01	Hyperarid
Tzaneen		130.55	10.29	7.88	0.02	Arid
Blouberg		141.73	10.59	7.47	0.01	Hyperarid
Eldorado		149.87	11.54	7.70	0.01	Hyperarid
Mokopane		132.84	11.46	8.63	0.01	Hyperarid
Polokwane		124.34	12.98	10.44	0.01	Hyperarid
Swartwater		154.93	11.42	7.37	0.01	Hyperarid
Zebediela		139.65	4.98	3.57	0.01	Hyperarid
ZZ2 Packhouse		132.60	9.79	7.38	0.01	Hyperarid

**Table 4:** Mann–Kendall statistics for (a) Rainfall and (b) PET

(a) Rainfall										
Station		Kendall's tau	S	Var(S)	P-value (two-tailed)	α	Trend	Sen's slope		Significant
Blouberg		-0.07	-52	7367	0.552	0.05	Decreasing	-0.04		No
Eldorado		0.09	37	3142	0.521	0.05	Increasing	0.08		No
Giyani		-0.09	-41	3142	0.475	0.05	Decreasing	-0.11		No
Gravelotte		-0.36	-167	3462	**0.005**	0.05	Decreasing	-0.38		Yes
Hoedspruit		-0.22	-96	3141	0.090	0.05	Decreasing	-0.28		Yes
Letaba		0.25	149	4958	**0.036**	0.05	Increasing	0.26		Yes
Mokopane		0.15	87	4958	0.222	0.05	Increasing	0.10		No
Polokwane		-0.15	-187	14292	0.120	0.05	Decreasing	-0.06		No
Swartwater		-0.05	-21	3462	0.734	0.05	Decreasing	-0.02		No
Tzaneen		0.01	4	7367	0.972	0.05	Increasing	0.00		No
Zebediela		-0.12	-116	9775	0.245	0.05	Decreasing	-0.06		No
ZZ2 Packhouse		-0.08	-43	4550	0.534	0.05	Decreasing	-0.06		No
(b) PET										
Blouberg	0.429		352	7926.67	** < 0,0001**	0.05	Increasing		7.380	Yes
Eldorado	-0.002		-1	3461.67	1.000	0.05	Decreasing		-0.161	No
Giyani	-0.441		-205	3461.67	**0.001**	0.05	Decreasing		-5.124	Yes
Gravelotte	0.520		258	3802.67	** < 0,0001**	0.05	Increasing		7.835	Yes
Hoedspruit	0.116		54	3460.67	0.368	0.05	Increasing		1.561	No
Letaba	0.524		330	5390.00	** < 0,0001**	0.05	Increasing		7.528	Yes
Mokopane	0.086		54	5390.00	0.470	0.05	Increasing		0.826	No
Polokwane	0.431		549	15158.33	** < 0,0001**	0.05	Increasing		2.069	Yes
Swartwater	0.492		244	3802.67	** < 0,0001**	0.05	Increasing		7.200	Yes
Tzaneen	-0.031		-24	7366.67	0.789	0.05	Decreasing		-0.254	No
Zebediela	0.511		506	10450.00	** < 0,0001**	0.05	Increasing		4.304	Yes
ZZ2 Packhouse	0.217		129	4958.33	0.069	0.05	Increasing		1.498	No

According to Seneviratne et al. (2021), due to the expected changes in climate and global warming, the probability of compound climate extremes is also expected to increase and become more frequent. An example is the variability in rainfall over South Africa between the mid-1970s and early 1980s (Matimolane et al., 2022), which led to extreme drought events occurring in 1982/1983, 1991/1992, 2003/2004, and more recently in 2014–2016 (Byakatonda et al., 2018). Over these periods, the effects of drought may be compounded by the occurrence of heatwaves. Mbokodo et al. (2023) reported that the 1982/1983, 1991/1992, and 2014/2016 drought events were accompanied by high-frequency heatwaves. The combination of compound drought and heatwaves poses a significant threat to socioecological systems, resulting in heightened impacts on communities. Heat waves often result in increased energy demand for cooling systems and straining power grids. Compound droughts, coupled with heatwaves, intensify water scarcity, impact irrigation, and crop growth and direct health risks, cause heat-related illnesses, and increase the likelihood of heatstrokes; this is more prevalent than the impacts experienced during singular or individual extremes (Tripathy et al., 2023). If these erratic patterns continue, many rural communities in semiarid environments are expected to be disproportionately impacted by the water supply. Climate change will cause unpredictable rainfall patterns and elevated temperatures, especially in semiarid and arid areas such as Eldorado and Swartwater.

**Fig. 2:**
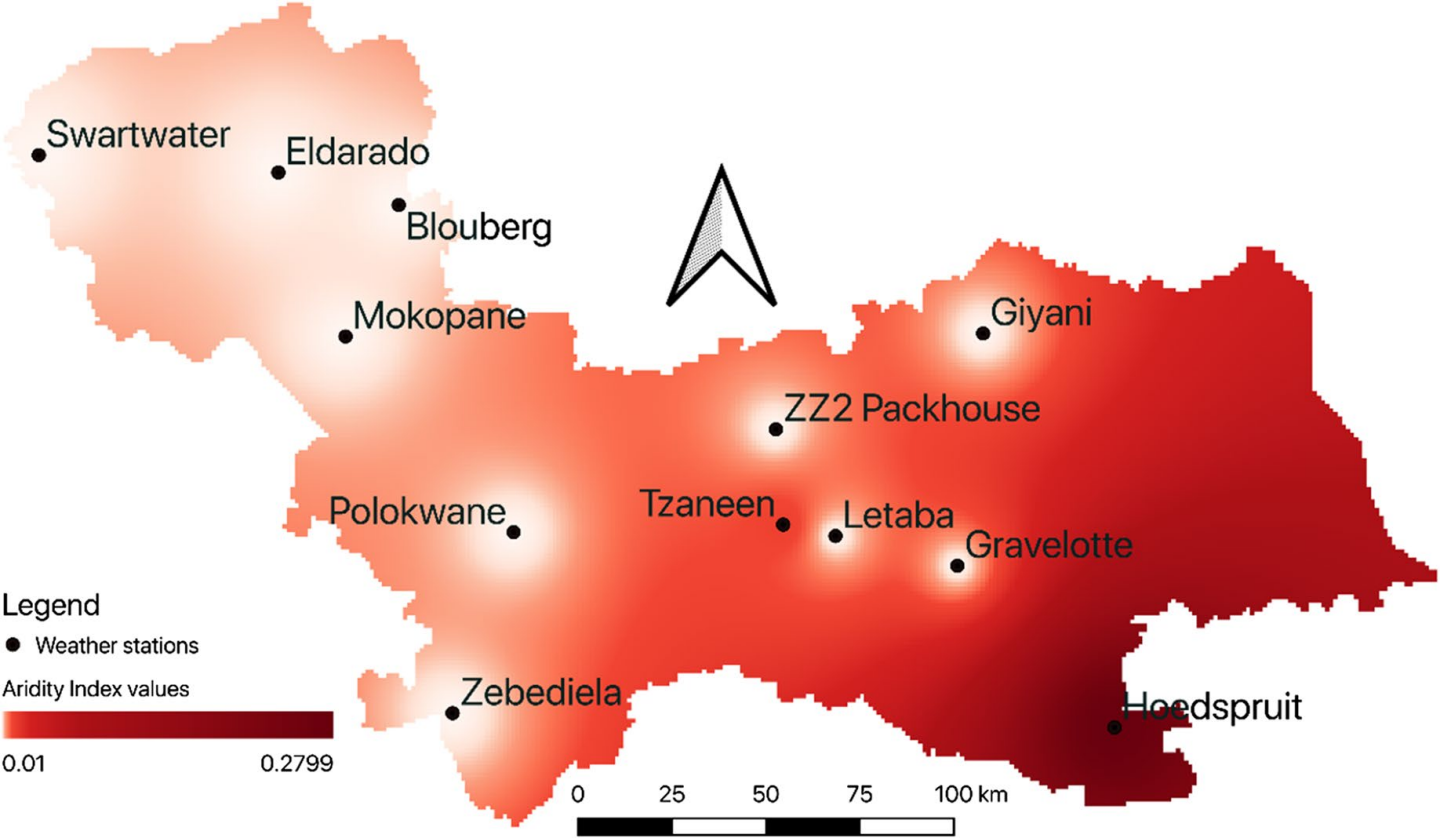
Spatial–temporal patterns of different stations (represented by black dots) aridity index over the Capricorn and Mopani district municipalities from 1990–2020

The spatial and temporal variations in climate conditions across the study area are presented in Fig. 2. The spatial distribution was determined using the inverse distance weighting (IDW) interpolation method (Chen et al., 2015) applied to the climate conditions shown in Table 3. IDW is widely used in spatial data interpolation and is considered to be a highly adaptable resource estimation method (Shahbeik et al., 2014). As shown in Fig. 2, aridity is more dominant in the west and decreases towards the east. The study area is characterized mainly by hyperarid conditions, with only two stations exhibiting arid and semiarid conditions, Tzaneen and Hoedspruit, respectively. This distribution is consistent with the temperature in the region, as the Swartwater station tends to record higher temperatures than the Tzaneen station.

### Drought occurrence and severity

**Fig. 3:**
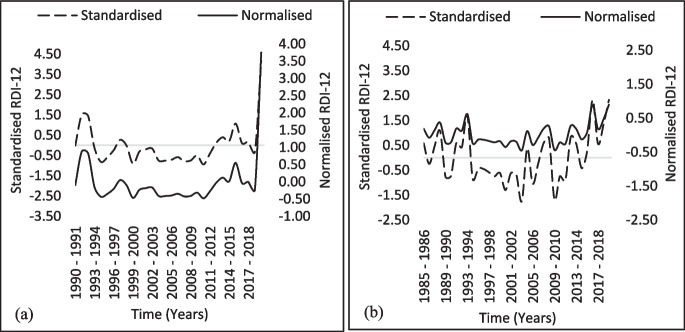
Monthly variation of RDI time series for Eldarado and Mokopane Stations: (**a**) Eldarado from 1990–1991 to 2019–2020 and (**b**) Mokopane from 1985–1986 to 2019–2020

**Fig. 4:**
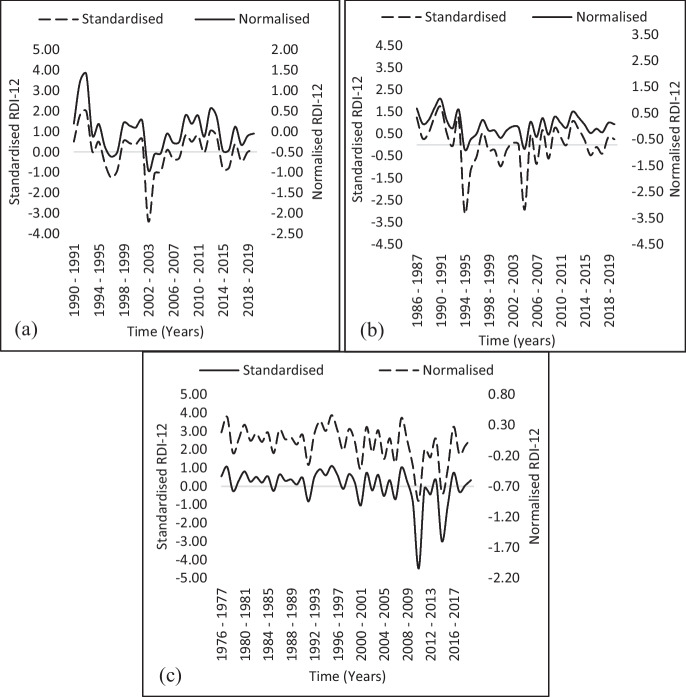
Monthly variation of RDI time series for Giyani, ZZ2 Packhouse and Zebediela Stations at the monthly time scale: (**a**) Giyani from 1990–1991 to 2019–2020; (**b**) ZZ2 Packhouse from 1986–1987 to 2019–2020; and (**c**) Zebediela from 1976–1977 to 2019–2020

Overall, the drought assessment revealed that the study area continuously experienced drought events across all drought classes as depicted by both the ${\text{RDI}}_{\text{st}}$ and ${\text{RDI}}_{\text{n}}$. Moderate drought conditions were prevalent with severe drought episodes less frequent, as shown in Figs. 3 and 4 The ${\text{RDI}}_{\text{st}}$ has been shown to indicate more intense drought conditions compared to the ${\text{RDI}}_{\text{n}}$. This is particularly evident in longer time scales where the cumulative effects of precipitation deficits and potential evapotranspiration are more pronounced. This is expected as the ${\text{RDI}}_{\text{st}}$ is derived by fitting a log-normal probability density function to the RDI data, which allows for a statistical comparison across different time scales and conditions. It categorizes drought severity into thresholds such as mild, moderate, severe, and extreme based on negative values, where lower values indicate more severe drought conditions(Thomas et al., 2016). On the other hand, the ${\text{RDI}}_{\text{n}}$ is calculated using the arithmetic mean of the RDI values over a specific period, which provides a simpler measure of drought conditions but may not capture the full severity as effectively as the standardized version (Shamsnia, 2014).

The Eldorado station in the CDM displayed the driest conditions over 30 years, with severe drought conditions persisting from 2000 to 2011 (Fig. 3a). The conditions were similar at Mokopane Station, as evidenced by the severe drought conditions experienced from 1995–2003 and punctuated by an alternating trend of near-normal conditions and extreme drought conditions in 2004 and 2010, as shown in Fig. 3b. The findings reveal a trend toward prolonged and severe drought periods. The prolongation of intervals between anticipated and varied rainfall patterns accompanied by a decrease in amount is a noticeable observation that can be attributed to changes in rainfall characteristics. This has a substantial impact on the availability and scarcity of water. These conditions have led to agricultural losses, diminished water supplies, widespread ecological stress, and increased pressure on local communities (Zwane, 2019). The occurrence of ‘moderate’ and ‘mild’ drought episodes is linked to decreased vulnerability to drought incidents. However, over the period under consideration, the station data revealed at least two extreme drought episodes, including several years of severe drought episodes, with the driest parts located primarily in the central and eastern regions of the study area.

Similarly, it was followed by 1996–1997 (-1.19) and 2003–2004, which had moderate drought conditions. However, the data for the Giyani station revealed continuous drought conditions during several periods, including 1995–1998 and 2014–2016, with the severity varying between mild and moderate drought conditions. While widespread severe droughts occurred from 2002–2003 across the study area, the RDI values revealed that several years experienced wet conditions, with RDI values of 2.15 in 1993 and 1.89 in 1992. As witnessed at the Eldorado, Giyani, Mokopane, and the ZZ2 Packhouse stations were also affected by extreme drought over the last two decades, i.e., 1994–1995 and 2004–2005 (Figs. 3a, b, 4a, and b). The station is located within a major agricultural area with over 3590 ha of production area, and the province contributes to as much as 66% of the total tomato production in the South African tomato industry (Tshiala & Olwoch, 2010). In the reported study, the authors highlighted the possible negative consequences of climate change on crop yields, with weeds and pests likely to increase. This is of particular concern for farmers who lack capital for research and development, advanced production technologies, and contemporary agricultural practices. In 2011, the Zebediela station had the highest RDI of -4.5 (Fig. 4c), indicating extreme drought conditions. This was shown to have lasted until 2016 (Nembilwi et al., 2021). The region is impacted by different drought severity episodes that extended throughout the meteorological period under consideration, and conditions close to normal were observed only in 2014.

The results of the 12-month RDI showed that 40% of the years (12 out of 30) under consideration experienced drought conditions of varying severity, with mild drought conditions observed in 50% of those years, highlighting the variability across the study area. The decreasing patterns in RDIs indicate the severity of drought occurrences in the region. The incorporation of PET in the RDI reinforces the concept that the severity of drought is affected by more complex factors, encompassing the increasing trends of PET and the interrelationship among temperature, humidity, and wind speed trends (Li et al., 2020). Due to the changing climate, persistent drought conditions point to the probability that semiarid areas will become hyperarid. The calculated RDI indices revealed that moderate and severe levels of drought severity were most frequently observed. In contrast, the extremely wet and extremely dry categories of drought severity had the least frequent occurrences in the RDI across the study area under the period of consideration.

### Climatic trends in the study area

Table 4 (a) and (b) show the MK and Sen slope statistics for rainfall and PET, respectively. The declining trends in rainfall and increasing PET based on temperature-based methods are further illustrated by the variability and high levels of PET across the municipalities over the years. The analysis revealed that 72% of the stations experienced decreasing rainfall trends, and two of these stations exhibited significant decreasing trends. For the case of PET, 92% of the stations show an increasing trend, with five showing a significant trend. From this analysis, it can be said that drought events in the study area are increasing in frequency, i.e., with drought events occurring in 2002/2003, 2005/2006, 2009/2010 and 2015/2016 amongst others. This is likely to negatively affect water resource availability because water recharge from rainfall is limited. The severity of drought is influenced by more intricate factors, including increasing trends in PET (Li et al., 2020).

According to the drought severity findings in this study, the study area is exposed to and has experienced extreme drought events with severe drought episodes. This finding supports those of Maponya (2012), who found that the province was prone to drought and its impacts from time to time, often coinciding with mature phases of the El Niño Southern Oscillation. The combined effect of all these changes in precipitation and temperature within the region will affect various sectors, notably in terms of hydrology, economics, and societal aspects, encompassing consequences for human activities, extensive crop failure, depleted groundwater resources, and a deficit in irrigation and drinking water (IEA, 2022). Given the expected variations in rainfall patterns and their variability within the context of a changing climate, the projected impact of climate change on water resource management underlines its significance for regions across all arid regions. The analysis revealed significant variability in PET patterns with low statistical uncertainty across the municipalities over the years. The analysis also revealed a significant positive PET trend across the 7 stations (Table 4b). This trend was only insignificant at the Letaba station, an area known to experience significant seasonal variation in monthly rainfall, which is located in a mountainous area and is covered by cropland (57%) and artificial surfaces (38%). The trend analysis results show an increase in the PET rate between 21 and 35 mm per decade.

**Fig. 5:**
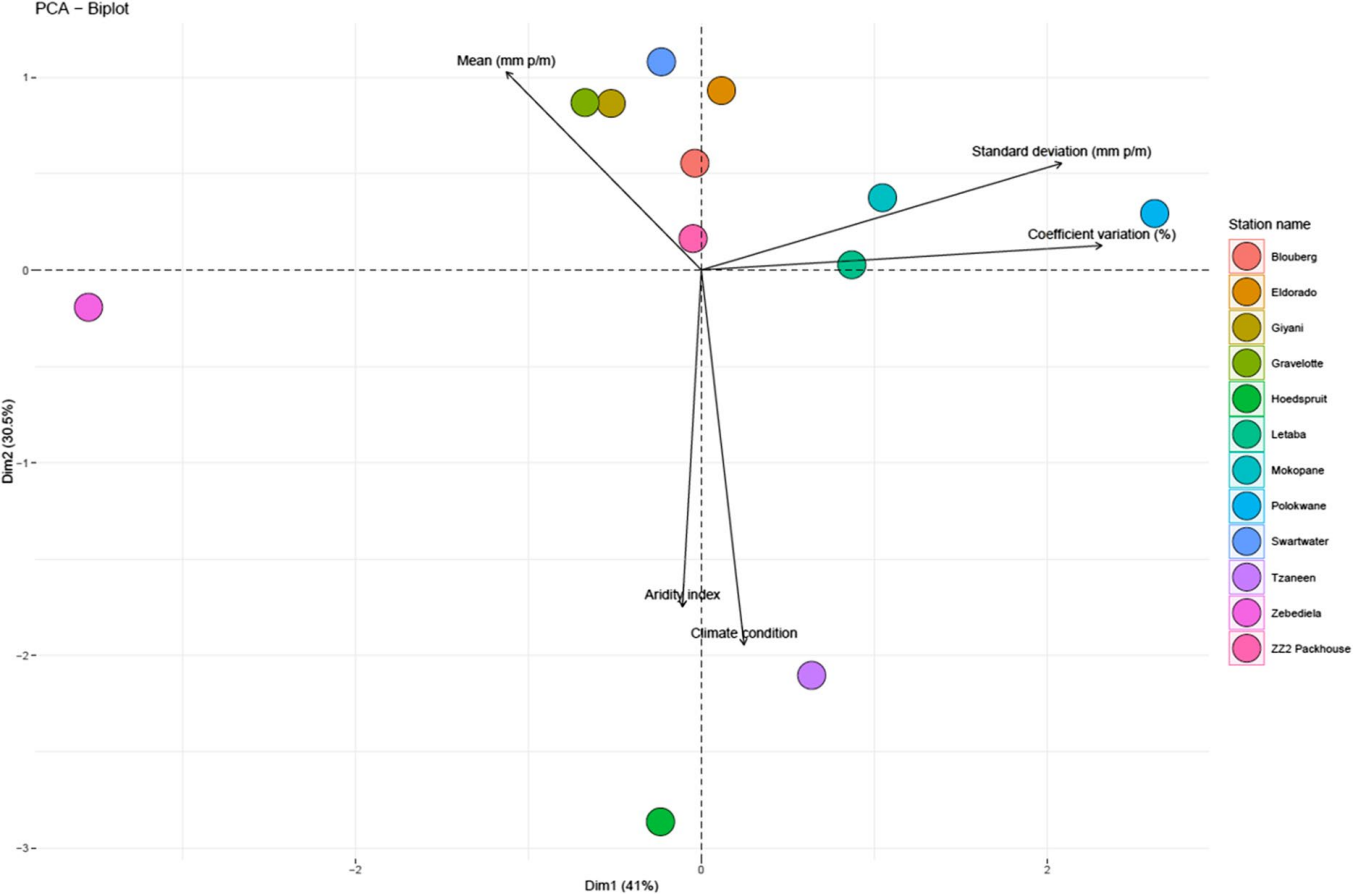
Biplot of Principal Component Analysis displaying the relationships between monthly aridity index and climatic variables at selected stations from 1970 to 2020. The first two principal components explain the majority of the variance, with arrows representing the climatic variables and points representing individual stations

To differentiate the monthly potential evapotranspiration (PET) and climate conditions at the selected weather stations, a principal component analysis (PCA) was performed. Figure 5 shows that the first two dimensions (Dim1 and Dim2) account for 71.5% of the total observed variation across the variables. The results demonstrate that the correlations among the stations are influenced by specific variables. Notably, stations located in hyperarid climates with low aridity indices formed distinct groups, separate from those in arid and semiarid climates (such as the Tzaneen and Hoedspruit stations) which have higher aridity indices.

The analysis reveals that climate conditions and aridity index variables are positively correlated, meaning that as the aridity index increases, the severity of climate conditions also increases. Conversely, mean monthly precipitation (mm, p/m) is inversely correlated with climate conditions, indicating that higher aridity is associated with lower levels of precipitation.

Based on these results, the climate in the region can be characterized as semiarid, as denoted by a general increase in several climatic indices, including minimum and maximum air temperatures and elevated levels of PET. These findings align with previous studies (Maponya, 2012; Rankoana, 2020; Tshiala et al., 2011), which report that parts of Limpopo Province are experiencing warming temperatures and are severely affected by dry spells that often escalate into severe drought due to the region's semiarid and arid climate. According to the aridity index, most of the study area falls within hyperarid, semiarid, or arid classifications (Fig. 5). Consequently, the likelihood of drought occurrence varies across different meteorological subdivisions, and the probabilities of drought can be further examined using the drought probability data (Bansode, 2014).

## Drought implications in the study area

The results of the study revealed that erratic rainfall, frequent dry spells and prevailing drought conditions are common in the study area and can adversely impact agricultural production and communities’ livelihoods. Additionally, these weather patterns directly impact water resources, which are crucial for the irrigation of crops, the maintenance of the environment, and human consumption. During drought years, there is a decline in agricultural activities, causing the agricultural sector to contribute less to the country’s gross domestic product (Orimoloye, 2022). Within the agricultural value chain, suppliers of inputs face reduced sales due to decreased demand, while agro-processors upstream experience a limited supply of produce, leading to higher costs due to scarcity. Ultimately, the impact of drought extends to consumers, as elevated food prices strain their budgets (Quiggin, 2007), particularly affecting those in financially vulnerable situations. As a result, the nation's food security is at risk since not everyone can dependably obtain an adequate quantity of affordable, nutritious food.

Drought has severe consequences for the agricultural sector in Limpopo Province, which utilizes approximately 60% of its freshwater resources (Nhamo et al., 2023). The province is particularly vulnerable to fluctuations in climatic conditions, often experiencing prolonged periods of dry spells leading to drought conditions (Maponya, 2012), which negatively affects a significant portion of agricultural production that relies on seasonal rainfall rendering (Andear, 2009; Maponya, 2012). As revealed through trend analysis for rainfall and PET, the decreasing trend of rainfall and elevated levels of PET is expected to increase the severity of droughts in the future due to climate change, making them a significant concern for the agricultural sector of the study area. In Limpopo Province, farmers are faced with increased financial liability from farming enterprises and are at risk of losing resources due to drought conditions (Schreiner et al., 2018). Baudoin et al. (2017) and Kuwayama et al. (2019) further emphasized that instances of inadequate water supply translate to the risk of lower agricultural yields. According to Trambauer et al. (2014), the situation in the province of Limpopo is not much better; previous severe droughts have caused crop failures and financial losses in the region.

Drought often results in water scarcity, presenting challenges for communities to meet their domestic water demands. In the study area, households face inadequate access to water due to inefficient infrastructure. In an earlier study in rural Limpopo Province, Matimolane et al. (2023) reported that up to 64% of households actively engaged in rainwater harvesting to augment the supply of water for domestic purposes. The severity of droughts has risen in recent decades, as evidenced by the trend analysis in this study together with the dry conditions experienced at the Eldorado and Mokopane stations from 2000–2011 and 1995–2003, respectively. Such persistent conditions may decrease the availability of water. According to Łabędzki (2016) and Williams et al. (2018), the consequences of droughts are anticipated to negatively impact water quality and diminish the amount of water accessible to vulnerable communities. This is particularly true given that the province is predominantly rural, with more than 80% of the population living in rural areas characterized by no development of water infrastructure, as noted by Malatji (2020).

In a region such as Limpopo Province, which relies heavily on agriculture and many livelihoods are tied to farming, drought can lead to economic instability and poverty (Mera, 2018). Farmers may struggle to provide for their families, leading to increased vulnerability and overall economic stability (Ndlovu, 2011), especially among marginalized communities. Additionally, drought can lead to environmental degradation, affecting ecosystems, biodiversity, and natural resources (Kuhnlein & Chotiboriboo, 2022). This, in turn, can impact livelihood practices that rely on these resources, such as fishing and forestry and the utilization of indigenous food.

## Conclusion and recommendations

This study examined drought severity in Limpopo Province over a 30 + -year period. Each year, climate data were collected for the same season from October to September, and PET was estimated using DrinC software. The Mann–Kendall and Sen’s slope estimator trend tests were used to assess the statistical significance of trends in annual climate extreme indices. Understanding the severity of drought events is vital to the management of their impacts, which have been shown to have far-reaching consequences. In addition to the immediate challenges faced by the agriculture and water supply sectors, there are socioeconomic repercussions, including food insecurity, economic instability, and potential displacement of vulnerable populations. The assessment of drought severity presented in this study provides valuable insights into the complex interplay of climatic factors, hydrological processes, and socioeconomic vulnerabilities. The findings reveal a trend of prolonged and intensified drought periods occurring over the study period, which is consistent with previous studies in 1982/1983, 1991/1992, 2003/2004, and more recently in 2014/2016. Severe and prolonged drought events are characterized by significant variability in rainfall, which negatively impacts water resource availability and causes widespread socioeconomic and ecological stress.

The RDI and trend analysis results revealed that the area experienced extreme drought in 2002/2003, with several years experiencing severe drought episodes. These conditions have led to agricultural losses, diminished water supplies, and increased pressure on local communities as they seek mitigation measures against the impacts of droughts. Therefore, considering the findings of this study, it is vital to continuously monitor and assess drought in drought-prone areas using new and improved methodologies, such as the RDI, which considers two significant components of the hydrological cycle. Continuous monitoring makes it possible to mitigate drought impacts among vulnerable communities in the face of a changing climate.

## Data Availability

The datasets used during the current study are available from the corresponding author upon reasonable request. Additional climate data are available upon request from the ARC-Natural Resources and Engineering Agrometeorology Programme. https://www.arc.agric.za/arc-iscw/Pages/Climate-Monitoring-Services.aspx.

## Declarations

All authors have read, understood, and complied as applicable with the statement on “Ethical responsibilities of Authors” as found in the “Instructions for Authors.”
